# Posttranslational Modifications, Localization, and Protein Interactions of Optineurin, the Product of a Glaucoma Gene

**DOI:** 10.1371/journal.pone.0009168

**Published:** 2010-02-11

**Authors:** Hongyu Ying, Xiang Shen, BumChan Park, Beatrice Y. J. T. Yue

**Affiliations:** Department of Ophthalmology and Visual Sciences, University of Illinois at Chicago College of Medicine, Chicago, Illinois, United States of America; Johns Hopkins University, United States of America

## Abstract

**Background:**

Glaucoma is a major blinding disease. The most common form of this disease, primary open angle glaucoma (POAG), is genetically heterogeneous. One of the candidate genes, optineurin, is linked principally to normal tension glaucoma, a subtype of POAG. The present study was undertaken to illustrate the basic characteristics of optineurin.

**Methodology/Principal Findings:**

Lysates from rat retinal ganglion RGC5 cells were subjected to N- or O-deglycosylation or membrane protein extraction. The phosphorylation status was evaluated after immunoprecipitation. It was found that while phosphorylated, optineurin was neither N- nor O-glycosylated, and was by itself not a membrane protein. RGC5 and human retinal pigment epithelial cells were double stained with anti-optineurin and anti-GM130. The endogenous optineurin exhibited a diffuse, cytoplasmic distribution, but a population of the protein was associated with the Golgi apparatus. Turnover experiments showed that the endogenous optineurin was relatively short-lived, with a half-life of approximately 8 hours. Native blue gel electrophoresis revealed that the endogenous optineurin formed homohexamers. Optineurin also interacted with molecules including Rab8, myosin VI, and transferrin receptor to assemble into supermolecular complexes. When overexpressed, optineurin–green fluorescence protein (GFP) fusion protein formed punctate structures termed “foci” in the perinuclear region. Treatment of nocadazole resulted in dispersion of the optineurin foci. In addition, tetracycline-regulated optineurin-GFPs expressing RGC5 stable cell lines were established for the first time.

**Conclusions/Significance:**

The present study provides new information regarding basic characteristics of optineurin that are important for future efforts in defining precisely how optineurin functions normally and how mutations may result in pathology. The inducible optineurin-GFP–expressing cell lines are also anticipated to facilitate in-depth studies of optineurin. Furthermore, the demonstrations that optineurin is an aggregation-prone protein and that the foci formation is microtubule-dependent bear similarities to features documented in neurodegenerative diseases, supporting a neurodegenerative paradigm for glaucoma.

## Introduction

Glaucoma, one of the leading causes of irreversible blindness worldwide [1], is characterized by progressive loss of retinal ganglion cells (RGCs) and axons, as well as distinctive cupping of the optic nerve head. The most common form of this disease, primary open angle glaucoma (POAG), is age-related and is frequently associated with elevated intraocular pressure.

Recent studies have demonstrated that POAG is genetically heterogeneous, caused by several susceptibility genes and perhaps also environmental factors [1]–[4]. Currently, a total of 14 chromosomal loci, designated as GLC1A to GLA1N, have been linked to POAG. Three candidate genes identified within these loci include myocilin (GLC1A), optineurin (GLC1E), and WD40-repeat36 (WDR36, GLC1G) [1]–[3]. Among them, optineurin is linked principally to normal pressure or normal tension glaucoma [5], a subtype of POAG. Optineurin mutations were noted to vary with ethnic background [6]. The Glu50Lys (E50K) mutation, found in Caucasian and Hispanic populations [6], seems to be associated with a more progressive and severe disease in normal tension glaucoma patients [7].

The human optineurin gene codes for a 577-amino acid protein that contains multiple coiled-coil domains and a C-terminal zinc finger [8]. The optineurin protein from different species has high amino acid homology [9] and the amino acid 50 glutamic acid residue is conserved in mouse, rat, chicken and cow [10]. Optineurin is ubiquitously expressed in non-ocular tissues such as the heart and brain [8] and in ocular tissues including the retina, trabecular meshwork, and non-pigmented ciliary epithelium [9]. In the retina, RGCs are immunolabeled with high intensity [10], [11].

Optineurin shares 53% amino acid homology with NEMO (NF-κB essential modulator) and was identified as a NEMO-related protein [12]. In murine pre-B and mouse T-cell hybridoma cell lines, optineurin expression was upregulated by tumor necrosis factor α (TNFα) and interferon and its phosphorylation was induced upon phorbol 12-myristate 13-acetate (PMA) stimulation [12]. More recently, optineurin has been shown to be a negative regulator of NF-κB [13]. There appears to be a negative feedback loop; the optineurin promoter activity and gene expression are elevated by treatment of TNF-α via the NF-κB pathway, and the optineurin induced in turn inhibits the NF-κB activation, dampening the TNFα signaling [13], [14].

Optineurin has in addition been shown to interact with transcription factor IIIA [15], Rab8 [16]–[20], huntingtin [16], myosin VI [17]–[19], metabotropic glutamate receptor [21], and TANK-binding kinase 1 [22]. It has been noted that optineurin may form complexes with Rab8 and myosin VI [17]–[20], mediating the targeting of myosin VI to the Golgi complex and may be involved in organization of the Golgi apparatus [17], [18]. siRNA studies also suggested a role of optineurin in the exocytosis of vesicular-stomatitis-virus G protein [18].

Despite these efforts, there is a surprisingly lack of data regarding the fundamental properties of optineurin protein. It is still unclear, for example, whether optineurin is itself associated with membranes, whether it is glycosylated, or whether it is indeed localized to the Golgi apparatus. Also there has been no illustration so far as to the phosphorylation status of optineurin. Neither has direct evidence been provided that optineurin forms complex with interacting proteins.

The present investigation was undertaken to elucidate the basic characteristics of optineurin in RGC5 and retinal pigment epithelial (RPE) cells. Specifically, the glycosylation and phosphorylation status, the possible Golgi localization, the membrane association, and the turnover rate of optineurin were investigated. The oligomerization of optineurin with itself, and the complex formation with Rab8, myosin VI, and transferrin receptor (TfR) were demonstrated for the fist time. In addition, optineurin was found to accumulate to form granular structures or foci in perinuclear regions when overexpressed and the foci formation was microtubule dependent.

## Results

### Glycosylation and Phosphorylation of Optineurin

From GenBank's reference sequence of human optineurin, the predicted molecular weight is 65921.9. However, on sodium dodecyl sulfate-polyacrylamide gel electrophoresis (SDS-PAGE) and by Western blotting, we consistently detected the endogenous optineurin at a higher, 74 kDa molecular size. The difference between the actual and predicted molecular weight led us to suspect that optineurin is posttranslationally modified.

**Figure 1:**
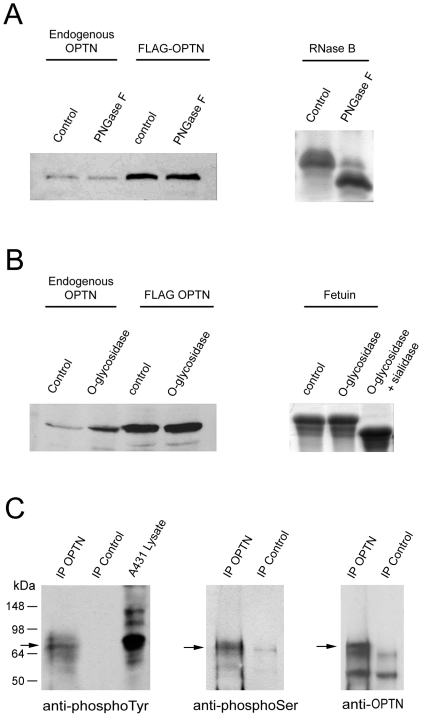
Optineurin is neither N- (A) nor O- (B) glycosylated but is phosphorylated (C). Lysates from RGC5 cells or those transfected with pOPTN-FLAG were untreated (control), or treated with PNGase F (for N-deglycosylation, **A**) or O-glycosidase (for O-deglycosylation) without (**B**) or with sialidase (not shown). The digested samples were subjected to Western blotting using anti-optineurin. The band pattern of optineurin (**A** and **B**, left panel) was not affected by any of the enzyme treatments. By Coomassie blue staining, the purified RNase B, as a positive control, had a band shift from 17 to 15 kDa after N-deglycosylation (**A**, right panel). The purified fetuin had a band shift from 64 to 55 kDa (**B**, right panel) after O-glycosidase and sialidase digestion. Fetuin was resistant to a single O-glycosidase digestion due to the fact that its N-linked and O-linked oligosaccharides are sialylated. **C**. Lysates from RGC5 cells were immunoprecipitated with either rabbit anti-C-terminal optineurin polyclonal antibody (IP OPTN) or normal rabbit IgG as an IP negative control (IP Control)) and were then immunoblotted with mouse anti-phosphotyrosine (anti-phosphoTyr), anti-mouse phosphoserine (anti-phosphoSer), or anti-C-terminal optineurin (anti-OPTN). The anti-optineurin but not rabbit IgG immunoprecipitated proteins were immunoreactive toward anti-phosphotyrosine (left panel) and anti-phosphoserine (middle panel). No immunoreactivity was observed with anti-phosphothreonine (not shown). Lysate from A431 (human epithelial carcinoma) cell line was used a positive control in anti-phosphotyrosine experiments. Immunoprecipitated pull down was verified by immunoblotting with anti-optineurin (anti-OPTN, right panel). Size markers are indicated. Arrows denote the 74-kDa optineurin band.

Several potential N-glycosylation sites were predicted for both human and rat sequences using the NetNGlyc 1.0 Server (http://www.cbs.dtu.dk/services/NetNGlyc/). One potential O-glycosylation site (202 T) was predicted in human optineurin using the DictyOGlyc 1.1 Server (http://www.cbs.dtu.dk/services/DictyOGlyc/). Lysates from RGC5 cells were treated with peptide N-glycosidase F (PNGase F, for N-deglycosylation) or O-glycosidase (for O-deglycosylaton) without or with α-2(3,6,8,9)-neuraminidase (sialidase), and Western blotted for optineurin. The band pattern of optineurin (Fig. 1A and B, left panel) was found not affected by any of these enzyme treatments. In parallel, the positive controls, ribonuclease B (RNase B) and fetuin, showed a band shift after N- or O-deglycosylation respectively (Fig. 1A and B, right panel). The former shifted from 17 to 15 kDa and the latter from 64 to 55 kDa with O-glycosidase and sialidase treatment. Fetuin was resistant to single O-glycosidase digestion due to the fact that its N-linked and O-linked oligosaccharides are sialylated [23]. A similar lack of glycosylation result was also obtained using lysates from RGC5 cells transfected with pTarget-FLAG-optineurin (OPTN) (Fig. 1A and 1B).

There is one site predicted (amino acid residue 356 for human and 361 for rat) in the optineurin sequence for tyrosine phosphorylation, 11 for threonine phosphorylation and at least 19 sites predicted for serine phosphorylation (*NetPhos 2.0 Server*). Phosphorylation experiment showed that optineurin immunoprecipitated from RGC5 lysates was immunoreactive against anti-phosphoserine and anti-phosphotyrosine (Fig. 1C), but not toward anti-phosphothreonine (data not shown). Immunoprecipitated pull down was verified by immunoblotting with anti-optineurin (anti-OPTN, Fig. 1C right panel).

### Localization of the Endogenous Optineurin

**Figure 2:**
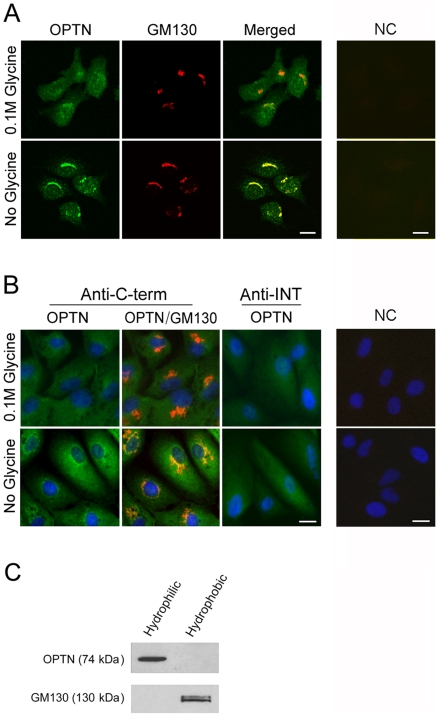
The endogenous optineurin is localized partially to the Golgi apparatus but is not a membrane protein. **A**. RGC5 cells were double immunostained with anti-C-terminal optineurin (in green) and anti-GM130 (Golgi marker, in red). The endogenous optineurin (OPTN) had a diffuse cytoplasmic distribution pattern when 0.1 M glycine was included in the PBS rinsing buffer (top, left panel). Without glycine (bottom, left panel), the optineurin staining was prominent in the perinuclear region, colocalizing (in yellow, Merged) with the Golgi apparatus. Cells incubated with only secondary antibodies as negative controls (NC) showed no staining (right panels). **B**. Human RPE cells were immunostained with anti-C-terminal (Anti-C-term, in green) or anti-INT (Anti-INT, in green) optineurin along with anti-GM130 (in red). The endogenous optineurin (OPTN) had a diffuse cytoplasmic distribution pattern when anti-INT optineurin antibody was used (middle right panels) regardless whether glycine was included in the initial rinse. Using anti-C-terminal antibody, the optineurin staining was more perinuclar and Golgi-associated without the glycine rinse (left panels). Cells incubated with only secondary antibodies as negative controls (NC) showed minimal staining (right panels). The nuclei were stained with DAPI in blue. Scale bar, 10 µm. **C**. Western blot analyses of hydrophilic and hydrophobic fractions from RGC5 cell lysates. Total proteins in RGC lysates were subjected to membrane protein extraction. The hydrophilic fraction (left lane) contained predominantly non-membrane cytosolic proteins and the hydrophobic fraction (right lane) contained membrane proteins. Optineurin (OPTN) was detected exclusively in the hydrophilic fraction. GM130, a Golgi marker used as membrane protein positive control, was detected in the hydrophobic fraction as expected.

RGC5 cells were double stained with anti-C-terminal optineurin and anti-GM130. The endogenous optineurin exhibited a diffuse cytoplasmic distribution pattern when 0.1 M glycine was included in the PBS rinsing buffer (Fig. 2A, top panels). Without glycine, the cytoplasmic staining was diminished and the optineurin appeared to be distributing more prominently in the perinuclear region, overlapping and colocalizing with the Golgi marker GM130 (Fig. 2A, bottom panels). Blocking by either 3% BSA or 10% normal goat serum made little difference in the optineurin staining patterns (photos not shown). Cells incubated with only secondary antibodies as negative controls showed no staining.

The optineurin localization in human RPE cells was re-examined. Previously, using anti-INT optineurin antibody and glycine-containing rinsing solution, it was noted that the endogenous optineurin had a diffuse staining pattern displaying little colocalization with the Golgi apparatus in human RPE and trabecular meshwork cells [17]. The new set of immunostaining was performed using both anti-C-terminal- and anti-INT-optineurin antibodies. It was found that anti-INT-optineurin yielded a diffuse, cytoplasmic staining with or without the glycine rinse (Fig. 2B). When anti-C-terminal optineurin was used, the staining pattern in RPE cells remained diffuse with, whereas it became more Golgi-associated without, the glycine wash (Fig. 2B).

### Optineurin Is Not a Membrane Protein

To determine whether optineurin itself is a membrane protein, lysates of RGC5 cells were subjected to membrane protein extraction. Proteins in the hydrophilic and hydrophobic fractions were immunoblotted with anti-C-terminal-optineurin and anti-GM130. The endogenous optineurin was seen to distribute exclusively in the hydrophilic fraction (Fig. 2C). GM130, used as positive control membrane protein, was predominantly detected in the hydrophobic fraction as expected (Fig. 2C).

### Optineurin Foci Formation upon Forced Exogenous Overexpression

**Figure 3:**
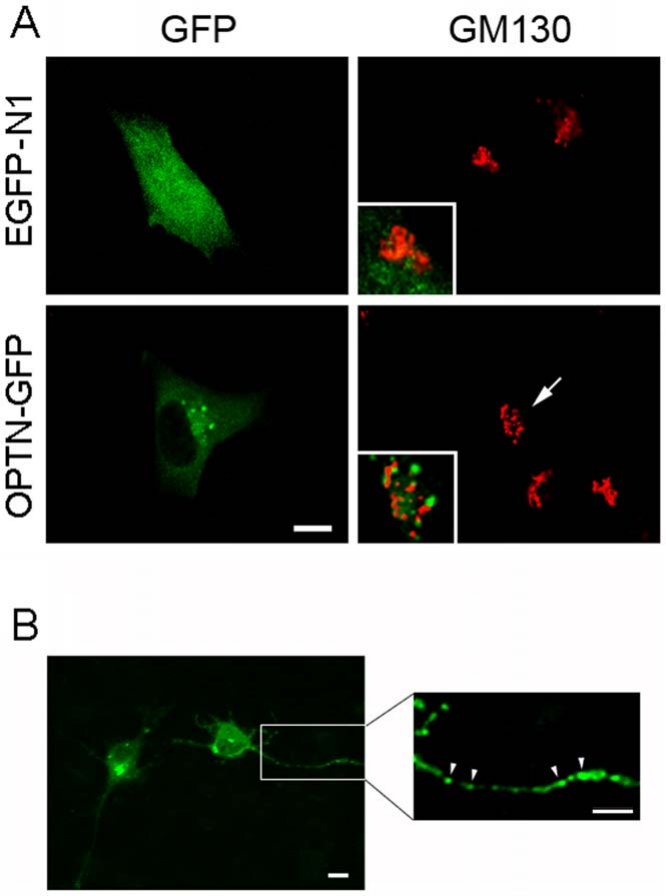
Cellular localization of overexpressed wild type optineurin. **A**. RGC5 cells were transfected for 20 h, fixed and immunostained. When transfected with pEGFP-N1 (mock control, top, left panel), green fluorescence was observed in the entire RGC5 cells including the nucleus. When transfected with pOPTN-EGFP (OPTN-GFP, bottom, left panel), diffuse green fluorescence was likewise seen in the cytoplasm of RGC5 cells. In addition, bright granular or punctuate structures termed foci were also noted in the perinuclear region around the Golgi apparatus (stained with anti-GM130 in red). Insets show the enlarged and merged images in the perinuclear region. Optineurin foci largely did not overlap with the GM130 staining. Golgi fragmentation in pOPTN-EGFP-transfected cells (arrows) was observed. Scale bar, 10 µm. **B**. RGC5 cells were transfected with pOPTN-EGFP for 20 h and underwent differentiation with treatment of 316 nM staurosporine for 4 h. Punctate optineurin-GFP foci (green) were found on the neurite extensions (arrowheads). The enlarged and amplified image of the blocked area is shown in the right panel. Scale bars, 10 µm.

When transfected with pEGFP-N1 (mock control), fluorescence from the expressed green fluorescence protein (GFP) was observed in the entire RGC5 cells including the nucleus (Fig. 3A, top left panel). When transfected with pOPTN-EGFP plasmid, diffuse green fluorescence from optineurin-GFP fusion protein was also seen in the cytoplasm of RGC5 cells. In addition, bright granular or punctuate structures in the perinuclear regions were noted (Fig. 3A, bottom left panel). These structures were very similar to those termed foci found previously in RPE cells [17]. Live cell imaging indicated that the foci were not stationary. They fused with or separated from each other (Video S1) and were mobile, sometimes exhibiting long distance movements (Video S2). When immunostained with Golgi marker GM130 in red (Fig. 3A, right panels), the optineurin foci in RGC5 cells did not colocalize with the Golgi apparatus (Fig. 3A, insert, bottom right panel). The lack of major colocalization, confirmed by confocal microscopy after sequential scanning, was also demonstrated previously in human RPE and trabecular meshwork cells [17]. Golgi fragmentation (Fig. 3A, arrow) was in addition noted in all these cell types [17].

Neurites were formed in RGC5 cells following treatment of staursporine [24]. In the differentiated, pOPTN-EGFP-transfeced RGC5 cells, punctate optineurin-GFP foci were found on the neurite extensions (Fig. 3B). Immunofluorescence of differentiated, nontransfected RGC5 cells also confirmed the presence of endogenous optineurin in neurites (data not shown).

### Distribution of Optineurin Foci Is Microtubule Dependent

**Figure 4:**
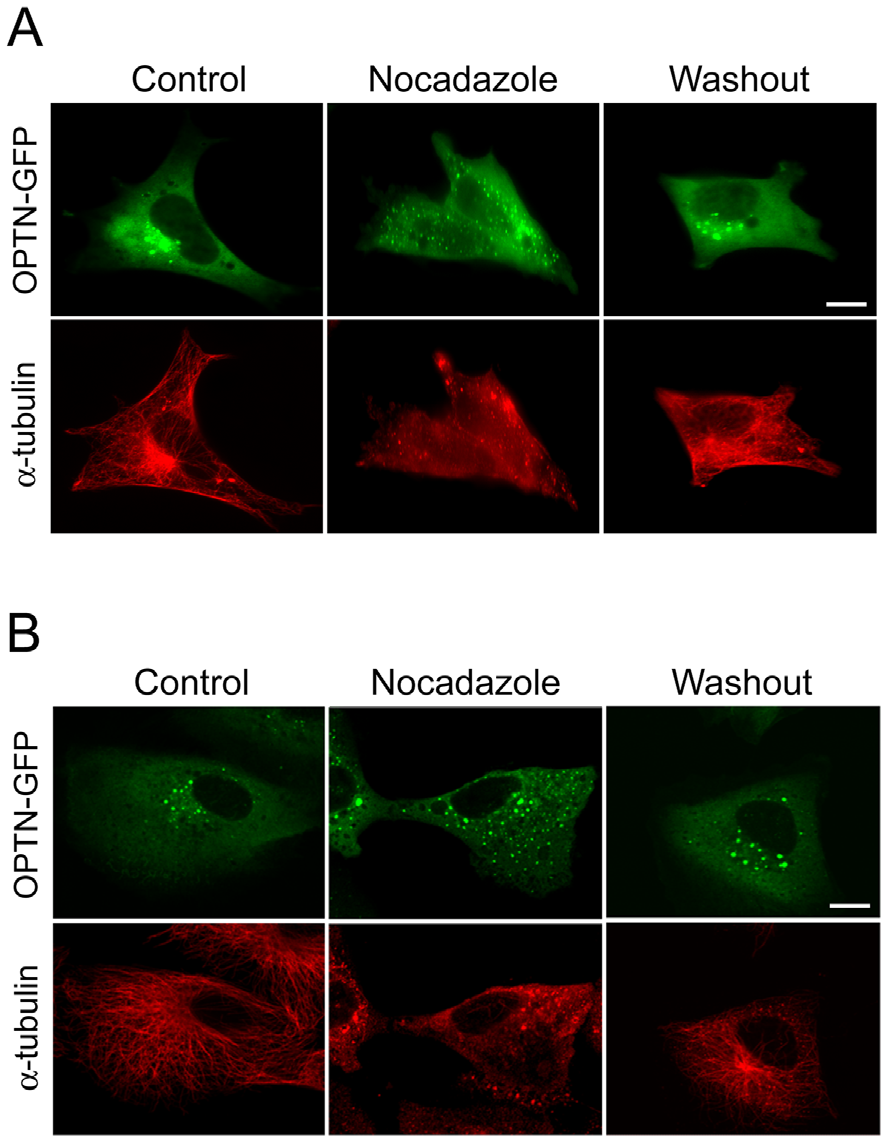
Distribution of optineurin foci is microtubule dependent. RGC5 (**A**) and RPE (**B**) cells expressing optineurin-GFP (OPTN-GFP) were untreated (Control) or treated with 10 µM nocodazole for 30 min. The cells were washed thoroughly to remove nocodazole (Washout) and allowed to recover for 1 h. The optineurin-GFP foci appeared in green and the microtubule network was visualized by immunostaining with anti-α-tubulin in red. The microtubule was disrupted upon nocodazole treatment and the optineurin foci were dispersed from perinuclear region to distribute evenly in the cytoplasm. Upon nocadazole removal, the microtubule network was restored and the foci returned to the perinuclear area. Scale bar, 10 µm.

The long distance movement of optineurin foci (Video S2) suggested that their distribution might depend on the microtubule network. pOPTN-EGFP-transfected RGC5 (Fig. 4A) and RPE (Fig. 4B) cells were treated with nocadazole, an agent that interferes with microtubule polymerization. Disruption of the network by nocadazole was confirmed by α-tubulin staining (Fig. 4) and under this condition, the optineurin foci were dispersed from the perinuclear region to distribute evenly in the cytoplasm. When nocadazole was removed, the microtubule was recovered and the optineurin foci were again clustered around the perinuclear region, similar to that seen in the untreated controls.

### Oligomerization of Optineurin

**Figure 5:**
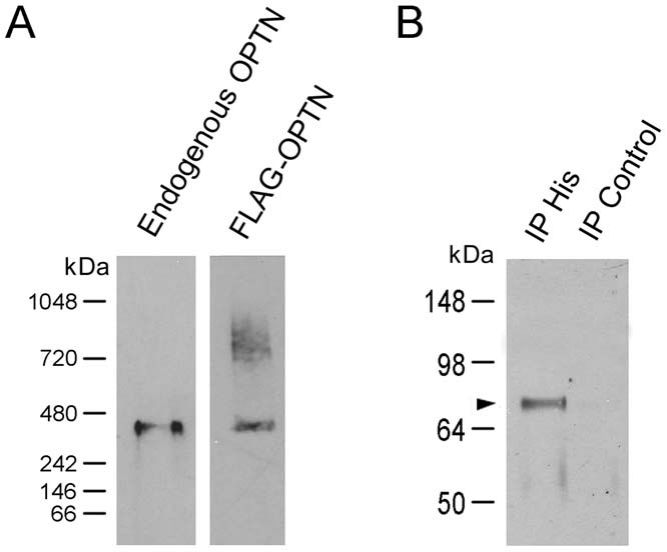
Oligomerization of optineurin. **A**. Native blue gel electrophoresis. Total protein from RGC5 cells (30 µg) and that (15 µg) from RGC5 cells transfected for 20 h to express FLAG-optineurin (FLAG-OPTN) fusion protein were subjected to native blue gel electrophoresis and Western blotting. The membranes were probed either with anti-optineurin to detect the endogenous optineurin (left lane) or anti-FLAG to detect the forced expressed FLAG-OPTN fusion protein (right lane). A band at approximately 420 kDa was observed in both cases in the native gel. With the FLAG-OPTN, higher molecular weight bands which may represent the complex formed with other optineurin binding partners were additionally seen. **B**. Co-immunoprecipiation. RGC5 cells were co-transfected with pTarget-FLAG-OPTN and pOPTN-His. Cell lysates were immunoprecipitated with rabbit anti-His antibody, and the immunoprecipitated proteins were probed with anti-FLAG antibody under reducing conditions. An immuoreactive optineurin band (arrowhead) was detected in the anti-His (IP His, left lane) but not in the rabbit IgG (IP Control, right lane) pull down. Size markers are indicated.

The secondary structure prediction based on the amino acid sequence of both human and rat optineurin showed that it is rich in α-helical coils (∼68%) (http://npsa-pbil.ibcp.fr/cgi-bin/npsa_automat.pl?page=npsa_gor4.html and http://www.russell.embl.de/cgi-bin/coils-svr.pl), which supercoil to form protein-protein interaction coiled coil domains [25]. To examine the possibility that optineurin can form oligomers or complexes, lysates from RGC5 cells were run on native blue gels followed by Western blotting for the endogenous optineurin under non-reducing conditions. A single band was detected at approximately 420-kDa, mass equivalent of homo-hexamer, indicating that optineurin interacted with itself to form hexamers (Fig. 5A, left lane).

Lysates from RGC5 cells transfected to express FLAG-optineurin fusion protein were also subjected to native blue gel electrophoresis and Western blotting. The 420 kDa-band was likewise observed using anti-FLAG (Fig. 5A, right lane). In addition, higher molecular weight bands which might represent complexes formed between FLAG-optineurin and other optineurin binding partners were also seen.

Co-immunoprecipitation (IP) experiments further proved that optineurin monomer could interact with another monomer to form complex. RGC5 cells were co-transfected to express N-terminal FLAG-tagged optineurin and C-terminal histidine (His)-tagged-optineurin. Thereafter, optineurin-His was pulled down with rabbit polyclonal anti-His antibody, and the pull down was immunoblotted under reducing conditions with mouse monoclonal anti-FLAG. As shown in Fig. 5B, the immunoprecipitate was immunoreactive to anti-FLAG (left lane). When normal rabbit IgG was used as the IP negative control (right lane), the FLAG-optineurin band (∼74 kDa, arrowhead) was not detected.

### Interaction of Optineurin with Other Proteins

**Figure 6:**
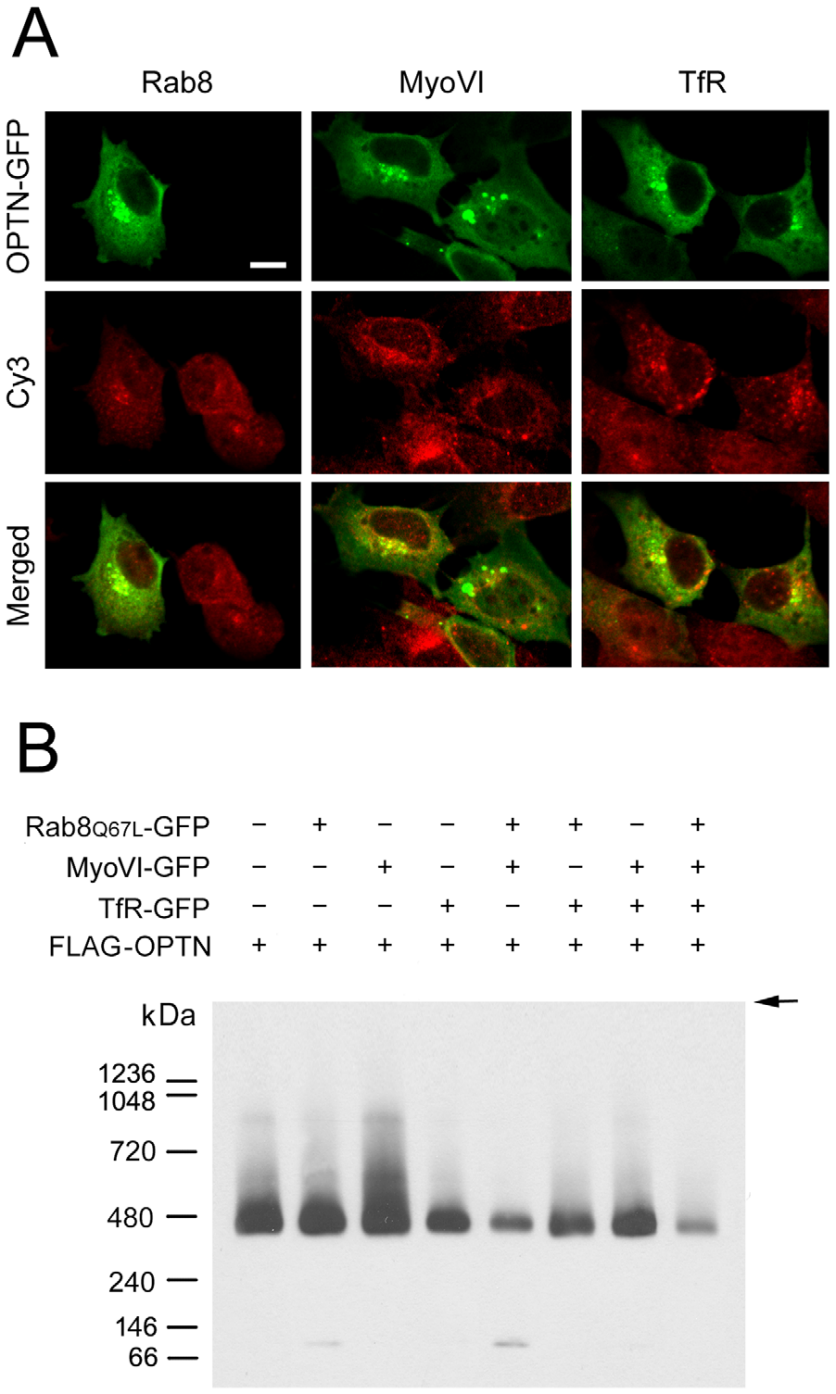
Protein interactions of optineurin. **A**. Colocalization of optineurin-GFP foci with Rab8, myosin VI (MyoVI) and transferrin receptor (TfR). RGC5 cells were transfected for 20 h to express optineurin-GFP (OPTN-GFP, in green) and immunostained with anti-Rab8, anti-MyoVI, or anti-TfR.Cy3-labled secondary antibody was used and the Rab8, MyoVI and TfR staining appeared in red (Cy3). Merged images are presented at the bottom panel. Colocalization of optineurin foci with Rab8, MyoVI, and TfR was observed in the perinuclear region in yellow in transfected cells. Those cells that stained positively with anti-Rab8, anti-MyoVI, and anti-TfR in red, but did not display any green fluorescence are non-transfected cells. **B**. Complex formation of optineurin with Rab8, MyoVI and TfR. RGC5 cells were co-transfected with p-Target-FLAG-OPTN (FLAG-OPTN) and pRab8_Q67L_-EGFP (Rab8_Q67L_-GFP), pMyoVI-EGFP (MyoVI-GFP), pTfR-EGFP (TfR-GFP), pRab8_Q67L_-EGFP + pMyoVI-EGFP, pRab8_Q67L_-EGFP + pTfR-EGFP, pMyoVI-EGFP+ pTfR-EGFP, or pRab8_Q67L_-EGFP + pMyoVI-EGFP + pTfR-EGFP. 20 µg of lysates from the transfected cells were subjected to native blue gel electrophoresis and Western blotting using anti-FLAG antibody. Super complexes with molecular sizes larger than 400 kDa were detected. The strongest complex formation was observed between FLAG-OPTN and MyoVI. The complex formation between them was reduced when Rab8_Q67L_ and/or TfR was present. They may compete with each other to interact with optineurin. Size markers are indicated. Arrow denotes the sample loading front.

Optineurin foci have been shown to colocalize, at least partially, with Rab8, myosin VI, and TfR in human RPE cells [17]. Such a result was reproduced in RGC5 cells (Fig. 6A). To investigate complex formation, RGC5 cells were co-transfected with pTarget-FLAG-OPTN and pRab8_Q67L_-GFP, pMyoVI-EGFP, and/or pTfR-EGFP in various combinations. By native blue gel electrophoresis and Western blotting, FLAG-optineurin was found to assemble into super molecular complexes with Rab8, myosin VI, and TfR (Fig. 6B). The molecular sizes of the complexes were larger than 400 kDa while monomer FLAG-optineurin could also be visualized in some of the combinations. The strongest complex formation appeared to be between FLAG-optineurin and myosin VI. It was somewhat reduced when Rab8_Q67L_ and/or TfR was present. When all four proteins were expressed in the cells, the complex formation seemed to be at its lowest level. Similar results were also found in RPE cells (data not shown).

### Establishment of Optineurin-GFP Tetracycline Regulated (Tet-on) RGC5 Stable Cell Lines

The inducible Tet-on optineurin-GFP expressing RGC5 stable cell lines were established using a single plasmid vector pTRE-OPTN-EGFP-INS-rtTA-IRES-hyg-pcDNA3.1z which contains both tetracycline regulatory and responsive components based on Clontech's Tet-on advance system. When transfected into RGC5 cells, optineurin-GFP was expressed upon doxycycline (Dox) induction.

**Figure 7:**
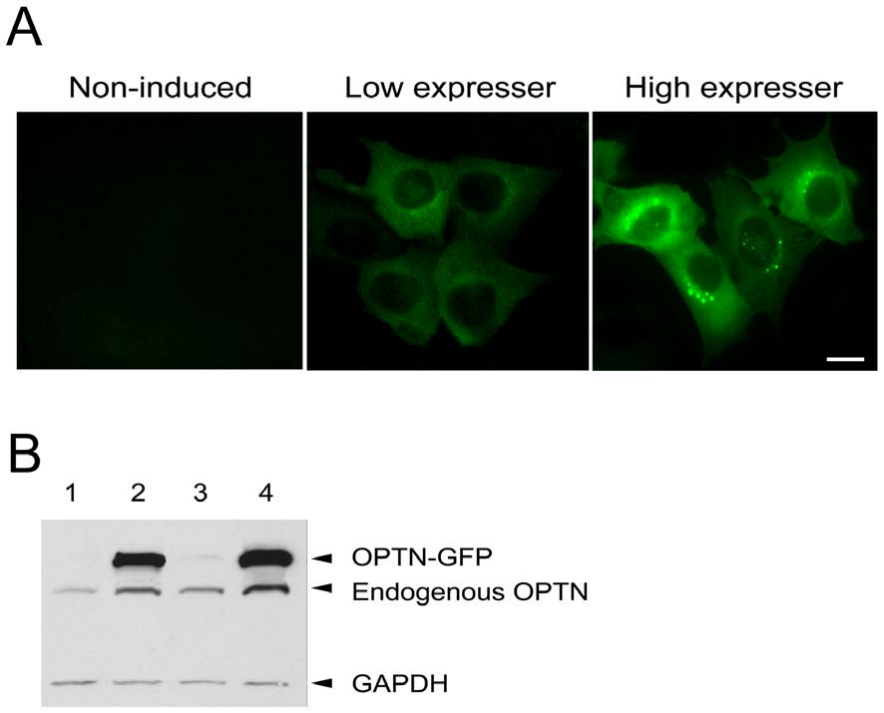
Inducible expression of optineurin-GFP in Tet-on RGC5 stable cell lines. **A**. Expression of optineurin-GFP by fluorescent microscope in two clones of the stable cell lines. Upon Dox induction, the low expresser (middle panel) showed a faint level of fluorescence in the cytoplasm while the high expresser (right panel) displayed bright fluorescence with foci observed in the perinuclear region. Non-induced control with background level of optineurin-GFP is presented on the left panel. **B**. Western blotting confirmed the expression of optineurin-GFP (OPTN-GFP) fusion protein in the low (lane 2) and the high (lane 4) expressers upon Dox induction. The optineurin-GFP band is barely visible without Dox induction (lane 1 for low expresser and lane 3 for high expresser). The endogenous optineurin (endogenous OPTN) band was seen in all 4 lanes. Glyceraldehyde 3-phosphate dehydrogenase (GAPDH) was used as a loading control.

After several rounds of selection, two clones of the inducible cells lines were obtained. One, the low expresser, exhibited a faint, while the other, the high expresser, showed a strong, green fluorescence after Dox induction (Fig. 7A). Western blotting also confirmed that the induced level of optineurin-GFP was low in the former but high in the latter (Fig. 7B). Microscopic examination of these stable cell lines indicated that the distribution of optineurin-GFP in the low expressers mimicked that of the endogenous optineurin, displaying a more spread out, cytoplasmic pattern whereas that in the high expresser mimicked what was seen in transfected cells, with foci observed in the perinuclear region.

### Turnover of Optineurin Protein

**Figure 8:**
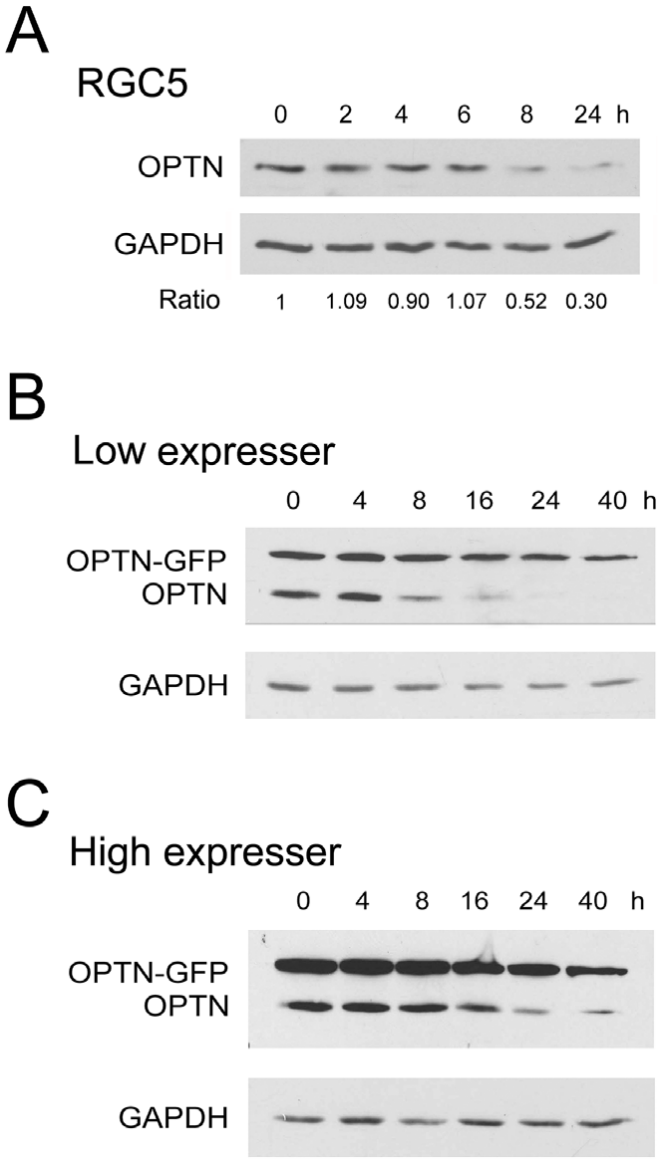
Turnover of endogenous and induced optineurin. **A.** Turnover of the endogenous optineurin in RGC5 cells. The cells were treated with cycloheximide (5 µg/ml) for 4 h to block the protein synthesis, and were harvested at the indicated time points. Western blotting for optineurin (OPTN) and glyceraldehyde 3-phosphate dehydrogenase (GAPDH, for loading control) was performed and densitometry was carried out. The ratios between the level of optineurin and that of GAPDH are presented. The half life of the endogenous optineurin was determined to be approximately 8 h. **B.** Turnover of optineurin-GFP (OPTN-GFP) in low expressers. **C.** Turnover of optineurin-GFP (OPTN-GFP) in high expressers. The cells were treated for 20 h with Dox (1 µg/ml) to express optineurin-GFP. After exposing to cyclohexmide for 4 h, the lysates were collected at the indicated time points. Anti-optineurin was used to detect both OPTN-GFP and the endogenous optineurin (OPTN). Compared to RGC5 endogenous optineurin (**A**), the induced optineurin-GFP had a delayed degradation rate in both low and high expressers. The half life of optineurin-GFP was approximately 20 h in low expressers. It was prolonged to >24 h in high expressers.

To study the turnover of the endogenous optineurin, RGC5 cells were treated with 5 µg/ml of cycloheximide for 4 h to block the protein synthesis, and were chased for 0, 2, 4, 6, 8, and 24 h. The level of the endogenous optineurin, judged by Western blotting, was decreased with time (Fig. 8A). The half life of the endogenous optineurin was calculated to be approximately 8 h.

To study the turnover of the overexpressed optineurin-GFP, the low and the high expresser clones were induced by Dox for 20 h, treated with cycloheximide, and chased for 0, 4, 8, 16, 24 and 40 h. As can be seen in Figs. 8B and 8C, the induced, exogenous optineurin-GFP still remained even after 40 h. The half life was approximately 20 h in the low expressers (Fig. 8B). The higher the optineurin-GFP expression, the more decelerated the degradation and the more prolonged was the half life (compare Fig. 8C versus 8B). The processing of the endogenous optineurin was also affected in the high expressers (Fig. 8C).

## Discussion

The present study provides information regarding basic characteristics of optineurin that are of fundamental importance. These properties have not been clearly illustrated previously in the literature. As one example, while potential glycosylation sites are predicted by computer analysis [26], it was never certain whether optineurin is a glycosylated protein. The current investigation is hence the first to establish that optineurin is neither N- nor O-glycosylated (Fig. 1A and B). In the field of optineurin, there was a controversy as to whether optineurin is secreted. Rezaie et al. [5] reported that optineurin was found in the aqueous humor and also in the culture medium, suggesting that it might be a secreted protein. However, a secretion assay conducted in our laboratory showed that optineurin is not secreted [17]. The lack of glycosylation demonstrated herein accords with the prediction that optineurin may not contain a signal peptide (http://www.cbs.dtu.dk/services/SignalP/) and provides support that optineurin is not a secreted protein. Both N-glycosylation and signal peptide sequences are documented to be crucial factors controlling constitutive protein secretion [27], [28].

In 2000, Schwamborn et al. [12] noted that the optineurin band was upshifted upon PMA treatment in E29.1 T cells, implying that optineurin might be phosphorylated, but the factual phosphorylation status was not addressed. Here we show that optineurin is serine- and tyrosine-, but not threonine-phosphorylated (Fig. 1C). Phosphorylation often introduces conformational changes and makes proteins more hydrophilic. In general, phosphorylation of proteins on serine, threonine and/or tyrosine is one of the significant regulatory mechanisms in gene expression and posttranslational modifications in both eukaryotes and prokaryotes. The phosphorylation status is known to be important for protein protein interaction and protein degradation. Tyrosine phosphorylation in particular has been recognized as a key means of signal transduction, implicated in cell growth, metabolic regulation and differentiation [29], [30]. A possibility of optineurin involvement in signal transduction may exist.

There have also been conflicting results as to optineurin's cellular localization at the Golgi apparatus. In a paper by Au et al. [31], it was somewhat vaguely stated that “optineurin appears to be a linker protein between myosin VI and Rab8, as all three proteins colocalize in the perinuclear region at/around the Golgi complex…”. Our immunostaining experiments provided clarifications that different staining patterns may result depending on the antibodies and washing procedures used. Two commercially available antibodies, anti-C-terminal- and anti-INT-optineurin, were tested. The former is reactive to human, mouse and rat optineurin while the latter is reactive only to the human protein. In both rat RGC5 and human RPE cells (Fig. 2), a diffuse, cytoplasmic distribution pattern of optineurin resulted when they were washed in glycine-containing solution and stained with anti-C-terminal-optineurin antibody. When glycine was omitted in the rinse, the cytoplasmic staining was low and optineurin seemed to locate largely in the perinuclear area overlapping with the Golgi marker GM130. Glycine has been used previously for antigen retrieval purposes [32]. Inclusion of glycine in the rinse might have uncovered antigen site(s), enhancing the optineurin staining in the cytoplasm and in turn rendering the Golgi association less distinctive. Anti-INT-human optineurin antibody, presumably reactive against epitope(s) different from those of anti-C-terminal antibody, yielded the diffuse, cytosolic pattern regardless whether glycine was used for the rinse in human RPE cells (Fig. 2B). We concluded unambiguously that optineurin has a diffuse, cytoplasmic distribution but a population of the protein is associated with the Golgi apparatus.

Because of the Golgi association, the next question was whether optineurin is itself a membrane protein. Subsequent experiments (Fig. 2C) revealed that optineurin is a hydrophilic, cytosolic protein, consistent with the prediction that the protein sequence contains no obvious transmembrane domains (http://www.cbs.dtu.dk/services/TMHMM/). The cytosolic localization of optineurin may accurately reflect its distribution and the role of optineurin is to interact with other proteins [19]. Sahlender et al. [18] suggested previously that optineurin may link myosin VI to Rab8 at the Golgi complex but did not specify or demonstrate experimentally whether optineurin is the Golgi anchor. Our findings stipulate that the optineurin-Golgi association is rather indirect and offer the insight that mostly likely it is Rab8 that serves as the Golgi anchor for optineurin, which then provides the next link for myosin VI.

Bright fluorescent granular or punctuate structures termed foci were observed (Fig. 3) when RGC5 cells were transfected to force overexpress optineurin-GFP. In agreement with our previous data on RPE and trabecular meshwork cells [17], Golgi fragmentation also seen in transfected RGC5 cells. The foci, located in the perinuclear region in close proximity to the Golgi apparatus, are not overtly associated with the organelle (Fig. 3A). It is of note that the optineurin foci in transfected RGC5 cells were also found upon differentiation in neurite extensions (Fig. 3B). Positive immunostaining of the endogenous optineurin was likewise observed in neurites in differentiated, nontransfected RGC5 cells (data not shown). These new observations suggest a possible physiological role of optineurin in polarized neurons, although additional studies are needed to substantiate this notion.

Optineurin has been shown to be present in the native RGCs in mouse eyes [11]. We also stained human, mouse, and rat retinal tissues with anti-optineurin, confirming its presence in RGCs (photographs not shown). The light microscopic examinations however, did not allow the determination whether optineurin is located at the Golgi apparatus or in dendritic trees. Immunogold studies will be necessary for such a determination.

The optineurin foci in transfected cells are likely formed by interactions of optineurin with itself and also with its interacting molecules. The self association of optineurin [33] and interaction between optineurin and Rab8 [16], myosin VI [18], and transferrin receptor [17], [31] have been documented previously via methods including yeast 2-hybrid and colocalization. In the article by Sahlender et al. [18], the authors suggested that “these (colocalization) results strongly indicate that myosin VI binds to and form a protein complex with optineurin *in vivo*”. Au et al. [31] stated that “these results, together with previous data, strongly suggest that myosin VI, optineurin, and Rab8 operate in the same basolateral sorting pathway and, most likely, form a functional complex.” Yet, the complex formation between these proteins has never been verified or experimentally demonstrated. Chalasani et al. [33] recently in a brief review also noted that “optineurin self-oligomerizes but the degree of oligomerization is not yet known”.

By native blue gel electrophoresis, we supplied evidence that optineurin is capable of forming 420 kDa homo-oligomers (Fig. 5A), which, based on the 67 kDa monomer size, is estimated to be hexamers. The optineurin foci were found to colocalize at least partially with Rab8, myosin VI, and TfR (Fig. 6A). Furthermore, optineurin associates with these proteins, either singly or in combination, to form supermolecular complexes with sizes larger than 400 kDa (Fig. 6B). There seemed to be also competitions in interactions with optineurin and we hypothesize that optineurin may need to be present in the cells in an optimal level such that all interactions are properly maintained. Disturbances of the various interactions, resulting from knock down or mutation, or when optineurin is in excess, might have pathophysiological consequences.

The foci formed by the overexpressed optineurin-GFP are dynamic structures. Visualized by live cell imaging, they move around in both short and long ranges (Videos S1 and S2). The long range movement probably involves the microtubule network. Subsequent experimentation proved that indeed the formation of optineurin foci is microtubule dependent (Fig. 4).

Furthermore, our laboratory established, for the first time, Tet-on inducible RGC5 cells lines using a single plasmid that contains both the tetracycline regulatory and response components. Establishment of Tet-on stable cell line normally involves two steps; first to generate the regulatory stable cell line, which is then used to produce the response stable cell line. In the regulatory cell line, the key component is a transactivator (rtTA-Advanced) that activates transcription from a tetracycline response element as a consequence of Dox treatment. In the response cell line, the key component is the gene of interest tightly controlled by a Ptight promoter. This two-step Tet-on system is not only time consuming but also reliant on the cell's sensitivity to at least two different selection antibiotics. Many ocular cells including RGC5 are very resistant to various commonly used selection antibiotics, including G418, zeocin, and puromycin. Fortunately, RGC5 cells are sensitive to hygromycin. Taking advantage of this sensitivity and using a single plasmid which requires only one round of cloning/selection made it feasible for establishment of the RGC5 stable cell lines. In this single plasmid, the responsive component is separated from the regulatory components with the 1.2 kb full length 5′-HS4 chicken β-globin insulator (INS) to block the potential cross talk between two expression cassettes [34]–[38].

Two constructs were ultimately created, with the two expression cassettes in either the same or the opposite orientation (Fig. S1). Both exhibited a low level of basal expression and high sensitivity to induction. With these constructs, two inducible cell lines were obtained; one expresses optineurin-GFP at low (low expresser) and the other at high (high expresser) levels upon Dox induction. In the low expressers, optineurin-GFP had a diffuse, cytosolic distribution (Fig. 7), comparable to that seen in the endogenous situation. In the high expressers, the foci were formed in the perinuclear region proximal to the Golgi (Fig. 7), similar to that observed in pOPTN-EGFP-transfected cells (Fig. 3A).

Turnover experiments were performed using RGC5 cells, as well as the low and high expressers. Previously, Schwamborn et al. [12] researched the half life of optineurin under PMA treatment. The investigators examined up to 5.5 h and concluded that optineurin has a rather long half life and that PMA treatment decreases its half life. The actual half life estimate however was not provided. We disclosed that the half life of the endogenous optineurin is approximately 8 h (Fig. 8A). When optineurin-GFP was expressed, the half life of the exogenous optineurin-GFP was increased to more than 20 h and the higher the expression level, the longer the half life. Meanwhile, the processing of the endogenous optineurin also appeared to be affected (Fig. 8C). The mechanisms underlying these intriguing findings are unclear. The inducible cell lines established are anticipated to provide a valuable model system for such studies and for others including gene expression alterations subsequent to optineurin overexpression.

RGC5, an immortalized RGC cell line established by transforming postnatal day 1 rat retinal cells with E1A adenovirus [39], has been used widely and extensively as a model of RGC for various investigations [24], [40]. It is of note however, that very recently a re-characterization of the RGC5 cells led to the revelation that they were in fact of mouse, not rat origin by both mitochondrial and nuclear DNA analysis [41]. The cells were not positive for known markers of ganglion cells such as neurofilaments or Thy1.2. Nevertheless, they did stain positively for neuronal markers β-tubulin and PGP9.5, as well as for the microtubule-associated protein, tau, and were able to differentiate with neurite extensions after treatment of staurosporine [24], [42] or trichostatin A [43], suggesting that these cells still represent neuronal precursor cells. We have repeated the results from RGC5 cells in RPE, human trabecular meshwork [17], and/or another neuronal rat pheochromocytoma PC12 [44] cells (data not shown).

Taken together, the current study provides new evidence and information, elucidating basic characteristics such as the glycosylation, phosphorylation and oligomerization status of optineurin. This information is important for further efforts in defining precisely how optineurin functions normally and how mutations result in pathology.

In neurodegenerative disorders such as Alzheimer's, Huntington's and Parkinson's, intracellular deposition of aggregated proteins into inclusion bodies, Lewy bodies, or aggresomes is a prominent cytopathological feature [45], [46]. The sequestation of proteins (such as α-synuclein in Parkinson's) into Lewy bodies is dependent on the microtubule-based dynein-dynactin pathway [47]. Mimicking at the molecular level, optineurin also appears to be an aggregate-prone protein with its foci formation contingent on the integrity of the microtubule network. These similarities further reinforce the notion that glaucoma shares common features with neurodegenerative diseases [48], [49] and support a neurodegenerative paradigm for glaucoma.

## Materials and Methods

### Cell Lines

Rat RGC5 cells were obtained from the University of Illinois at Chicago, Ophthalmology departmental core facility, deposited by Dr. Paul Knepper [50] and originally from Dr. Neeraj Agarwal [39]. In some experiments, RGC5 cells were differentiated with 316 nM staurosporine for 4 h [39]. Human RPE (ARPE 19) cells were from American Type Cell Culture (Manassas, VA). The cells were cultured in complete medium as previously described [17], [51].

### DNA Constructs

Optineurin expression vectors pTarget-FLAG-wild type optineurin (OPTN) and pOPTN-EGFP, as well as pRab8_Q67L_-EGFP with constitutively active Rab8 (Rab8_Q67L_) fused to EGFP were constructed as previously described [17]. pOPTN-His-pcDNA3.1z was made by adding a His tag to the C-terminal of optineurin by PCR using pOPTN-EGFP as the template and the subsequent subcloning of OPTN-His into EcoR V linearized pcDNA3.1z vector (Clontech, Mountain View, CA). pTfR-EGFP was made by PCR amplification of full length TfR gene from human MGC verified cDNA (clone ID 3354176, Open Biosystem, Huntsville, AL) and subcloned into pEGFP-N1 (EcoR I-BamH I). Human myosin VI full length fused to EGFP (pMyoVI-EGFP) was generously provided by Dr. Tama Hasson, University of California San Diego [52].

To generate inducible cell lines, a single Tet-on expression vector pTRE-OPTN-EGFP-INS-rtTA-IRES-hyg-pcDNA3.1z was created. Briefly, to produce pTRE-OPTN-EGFP construct, OPTN-EGFP fusion gene was digested from pOPTN-EGFP and cloned into EcoR I and BamH I linearized pTRE-tight vector (Clontech). To construct pIRES-hyg-pcDNA3.1z, IRES (internal ribosome entry site from virus EMCV) was digested from pBL-EP (kindly provided by Dr. John Olsen, University of North Carolina) by EcoR I and EcoR V and cloned into linearized pcDNA3.1z vector. Hygromycin was then shuttled from the linearized hygromycin marker (Clontech) to make pIRES-hyg-pcDNA3.1z. Next, the 1.2 kb fragment of rtTA driven by CMV promoter was digested with EcoR I and Hind III from plasmid pTet-on Advance vector (Clontech), blunt ended and ligated with BamH I linearized and blunt ended pIRES-hyg-pcDNA3.1z to result in prtTA-IRES-hyg-pcDNA3.1z. The 3 kb TRE-OPTN-EGFP fragment was then shuttled from pTRE-OPTN-EGFP to pBS II-SK(+) vector and the 1.2 kb fragment of 5′-HS4 chicken β-globin insulator (INS) was digested with Xba I from plasmid pJC13-1 (kindly provided by Dr. Gary Felsenfeld, National Institute of Diabetes and Digestive Diseases, Bethesda, MD) and ligated with Spe I linearized plasmid pBS-TRE-OPTN-EGFP to generate pBS-TRE-OPTN-EGFP-INS. Finally, the 4 kb fragment of rtTA-IRES-hyg was digested with Bgl II and Pvu II from plasmid prtTA-IRES-hyg-pcDNA3.1z, blunt ended and ligated with Sac II linearized and blunt ended vector pBS-TRE-OPTN-EGFP-INS to construct pTRE-OPTN-EGFP-INS-rtTA-IRES-hyg-pcDNA3.1z. Two different types of clones were selected with the two expression cassettes oriented either in same or the opposite direction (Fig. S1). Both clones, when transfected into RGC5 cells, induced optineurin-GFP expression upon Dox treatment.

### Immunohistochemistry

For localization of the endogenous optineurin, rat RGC5 without or with staurosporine treatment and human RPE cells were washed with rinsing solution, fixed, blocked, and double immunostained with rabbit polyclonal C-terminal or INT-optineurin antibody (1∶100, Cayman Chemical, Ann Arbor, MI) and mouse monoclonal anti-GM130 (1∶200, BD Biosciences, San Jose, CA). FITC-goat anti-rabbit and Cy3-goat anti-mouse IgG (1∶200, Jackson ImmunoResearch, West Grove, PA) were used as the secondary antibodies. RPE cells were stained with two different optineurin antibodies (C-terminal, reactive to both human and rat, and INT, reactive to human optineurin, amino acid residues 115–130) while RGC5 cells were stained with only the C-terminal antibody. Two different blocking solutions (3% bovine serum albumin (BSA) and 10% normal goat serum) and two rinsing solutions (phosphate buffered saline (PBS) with or without 0.1 M glycine, pH 8.2) were used for the staining procedure, and the staining results were compared side by side. In negative controls, the primary antibodies were omitted and the cells were incubated with only secondary antibodies. The slides were mounted in Vectashield (Vector Laboratories, Burlingame, CA) with 4′,6-diamidino-2-phenylindole (DAPI).

In optineurin overexpression studies, cells were transfected [51] for 20 h with pEGFP-N1 (mock control) or pOPTN-EGFP using Lipofectamine LTX transfection reagent (Invitrogen, Carlsbad, CA), fixed, and immunostained with mouse anti-GM130 (1∶200), anti-Rab8 (1∶200, BD Biosciences), anti-myosin VI (1∶200, Sigma, St. Louis, MO), or anti-TfR (1∶200, Zymed, South San Francisco, CA).

In some experiments, RGC5 and RPE cells transfected with pOPTN-EGFP for 20 h were untreated or treated with 10 µM nocodazole (Sigma) for 30 min. They were then washed thoroughly, and re-incubated in complete media for 1 h, fixed, and stained with mouse anti-α-tubulin (1∶200, BioGeneX, San Ramon, CA).

Photography was carried out using a 63× oil objective on an Axioscope (Carl Zeiss MicroImaging, Thornwood, NY) with the aid of Metamorph software (Molecular Devices, Downingtown, PA). In some experiments, confocal microscopic analysis was performed on a Leica SP2 confocal system (Leica Microsystems, Bannockburn, IL) using sequential scanning to minimize the bleed through.

### IP and Western Blotting

RGC5 cells were lysed with IP lysis buffer (250 mM NaCl, 50 mM Tris/HCl, pH 7.5, 5 mM EDTA, 0.5% Nonidet P40) supplemented with protease inhibitor cocktail (Sigma). Protein concentration was determined by bicinchoninic acid protein assay (Pierce, Rockford, IL). The lysate was immunoprecipitated with rabbit anti-optineurin (Bethyl Laboratories, Montgomery, TX) or rabbit normal IgG (negative control) using the Catch and Release kit (Millipore, Billerica, MA). The proteins pulled down were subjected to SDS-PAGE under reducing conditions. The proteins were transferred to nitrocellulose membrane using iBlot™ gel transfer device (Invitrogen). Phosphorylation of the proteins was assessed by immunoblotting with mouse monoclonal anti-phosphoserine, anti-phosphothreonine (1∶2,000, Sigma) and anti-phosphotyrosine (1∶1,000, PY20, BD Transduction laboratories, San Jose, CA). Pulled down proteins were also immunoblotted with rabbit anti-C-terminal optineurin antibody (1∶2000) to confirm that optineurin protein was immunorecipitated. Immunoreactive optineurin bands were detected by chemiluminescence using SuperSignal Substrate (Pierce).

Co-IP experiments were performed after co-transfecting RGC5 cells with pTarget-FLAG-OPTN and pOPTN-His-pcDNA3.1z for 20 h. Rabbit anti-His polyclonal antibody (Santa Cruz Biotechnology, Santa Cruz, CA) was used to immunoprecipitate optineurin-His fusion protein from the lysates. The pulled down proteins were then subjected to SDS-PAGE and Western blotting with mouse anti-FLAG monoclonal antibody (Strategene, La Jolla, CA).

### Membrane Protein Extraction

RGC5 cells (5×10^6^) were washed with PBS and lysed in IP lysis buffer. Total cell lysate was subjected to membrane protein extraction using Mem-PER Eukaryotic Membrane Protein Extraction Reagent kit (Pierce). Following a mild detergent-based protocol, the hydrophobic proteins were separated from the hydrophilic ones through a phase partitioning. Cross-contamination of cytosolic proteins into the membrane fraction is typically <10%. The separated hydrophobic and hydrophilic fractions were then subjected to SDS-PAGE and immunoblotted using rabbit anti-C-terminal-optineurin or mouse anti-GM130 (used as positive control for membrane protein).

### Glycosylation

Total protein (50–100 µg) from RGC5 cells or those transfected with pTarget-FLAG-OPTN was digested with PGNase F (0.25 units, Enzymatic In-Solution N-deglycosylation kit, Sigma) for N-deglycosylation or with O-glycosidase (2.5 mUnits, Sigma) without or with addition of sialidase A (5 mUnits, Sigma) for O-deglycosylation per manufacturer's instructions. Samples were subjected to SDS-PAGE and Western blotted with rabbit anti-C-terminal optineurin (1∶2,000) to detect the endogenous optineurin in RGC5 cells, or mouse anti-FLAG (1∶5,000) to detect FLAG-optineurin in transfected cells. The purified RNase B (Sigma) was used as a positive control in parallel in the N-deglycosylation experiment and the purified bovine fetuin (Sigma) as a positive control in the O-deglycosylation experiment. These gels were stained with Coomassie blue and scanned for documentation after SDS-PAGE.

### Native Blue Gel Electrophoresis

To examine the interaction of optineurin with itself, RGC5 cells or those transfected with pTarget-FLAG-OPTN were lysed in IP buffer and the lysates were subjected to native blue gel electrophoresis. Total protein was mixed with 2x native sample buffer (0.125 M Tris-HCl, pH 6.8, 20% glycerol, 0.01 mg/ml bromphenol blue) and resolved on NativePAGE™ Novex® Bis-Tris Gel (Invitrogen) under nonreducing conditions. Protein bands were transferred to nitrocellulose membrane and the endogenous native optineurin band(s) was detected using rabbit anti-C-terminal optineurin polyclonal antibody (1∶2,000) and horseradish peroxidase (HRP) conjugated goat anti-rabbit secondary antibody (1∶10,000, Jackson ImmunoResearch). FLAG-optineurin was detected using mouse anti-FLAG monoclonal antibody (1∶5000, Stratagene, La Jolla, CA).

To investigate complex formation, RGC5 and RPE cells were transfected either with pTarget-FLAG-OPTN alone or co-transfected with pRab8_Q67L_-EGFP, pMyoVI-EGFP, pTfR-EGFP, pRab8_Q67L_-EGFP + pMyoVI-EGFP, pRab8_Q67L_-EGFP + pTfR-EGFP, pMyoVI-EGFP + pTfR-EGFP, or pRab8_Q67L_-EGFP + pMyoVI-EGFP + pTfR-EGFP. Cell lysates were subjected to native blue gel electrophoresis and Western blotting. The native FLAG-optineurin bands were immunoprobed with mouse anti-FLAG monoclonal antibody (1∶2,000) and HRP conjugated goat anti-mouse secondary antibody (1∶10,000, Jackson ImmunoResearch).

### Screening and Cloning of Tet-on Optineurin-GFP Stable Cell Lines

RGC5 cells were transient transfected for 20 h with pTRE-OPTN-INS-hyg-IRES-rtTA-CMVp or pTRE-OPTN-INS-CMVp –rtTA-IRES-hyg construct in T-25 flasks using Lipofectamine LTX transfection reagent. The growth medium was changed to selection medium containing 100 µg/ml of hygromycin and the selection continued for about 2 weeks until medium sized colonies grew out. The cells were then trypsinized and induced with 1 µg/ml of Dox for 24 h. Bright GFP positive cells were sorted using DakoCytomation MoFlo into 96 well plates (1 cell/well) with maintenance medium (with 50 µg/ml of hygromycin but without Dox) for another 2 weeks. Cells were screened for high or low optineurin-GFP expression after induction with Dox by fluorescence microscopy. The protein level of optineurin-GFP was assessed by immunoblotting against polyclonal anti-optineurin and anti-glyceraldehyde 3-phosphate dehydrogenase (GAPDH, Trevigen, Gaithersburg, MD). Both high and low expressers were allowed to multiply and were subsequently banked in liquid nitrogen.

### Turnover of Optineurin

To determine the turnover rate of the endogenous optineurin, RGC5 cells were plated onto 6-well plates (1×10^6^ cells/well). After incubation with medium containing 5 µg/ml of cycloheximide (Calbiochem, Gibbstown, NJ) for 4 h, the cells were harvested at 0-, 2-, 4-, 6-, 8-, and 24-h time points similar to that described previously [53], [54]. Total protein (40 µg) from each sample was subjected to Western blotting using rabbit anti-C-terminal optineurin polyclonal antibody. The membrane was also immunoblotted for GAPDH as a loading control. Densitometry was performed. The band intensity of the endogenous optineurin was normalized to that of GAPDH. The half life of GAPDH is reported to be longer than 72 h [55].

The turnover of optineurin-GFP in Tet-on high and low RGC5 expressers were determined after induction for 20 h with Dox and a treatment for 4 h with cycloheximide. Proteins harvested from cells 0, 4, 8, 16, 24, and 40 h later were immunoblotted with anti-optineurin and anti-GAPDH. All experiments were repeated at least 3 times.

## Supporting Information

Figure S1 Single plasmid constructs with two expression cassettes. The two cassettes (responsive element, in yellow, and regulatory element, in blue) are separated by 5′-HS4 chicken β-globin insulator (INS, in pink). A shows the plasmid in which the regulatory element is of the same orientation as the responsive element. B shows the plasmid in which the regulatory element is of the opposite orientation as the responsive element.
(7.23 MB TIF)

Video S1 Movement of optineurin foci monitored by live cell microscopy. A live RGC5 cell transfected to express optineurin-GFP was imaged. Images were collected every 7 seconds for 7 minutes. Frame rate: 30 frames/2 seconds.
(2.58 MB AVI)

Video S2 Long-distance movement of optineurin foci. A live RPE cell transfected to express optineurin-GFP was imaged. Images were captured every 7 seconds for a total of 23 frames. Frame rate: 23 frames/second. A long-distance movement was seen (arrow).
(5.08 MB AVI)
