# Supplementary figures and images for: Posttranslational Modifications, Localization, and Protein Interactions of Optineurin, the Product of a Glaucoma Gene
Source: PLoS One. 2010 Feb 11;5(2):e9168. doi: 10.1371/journal.pone.0009168 (PMC2820081; doi:10.1371/journal.pone.0009168)

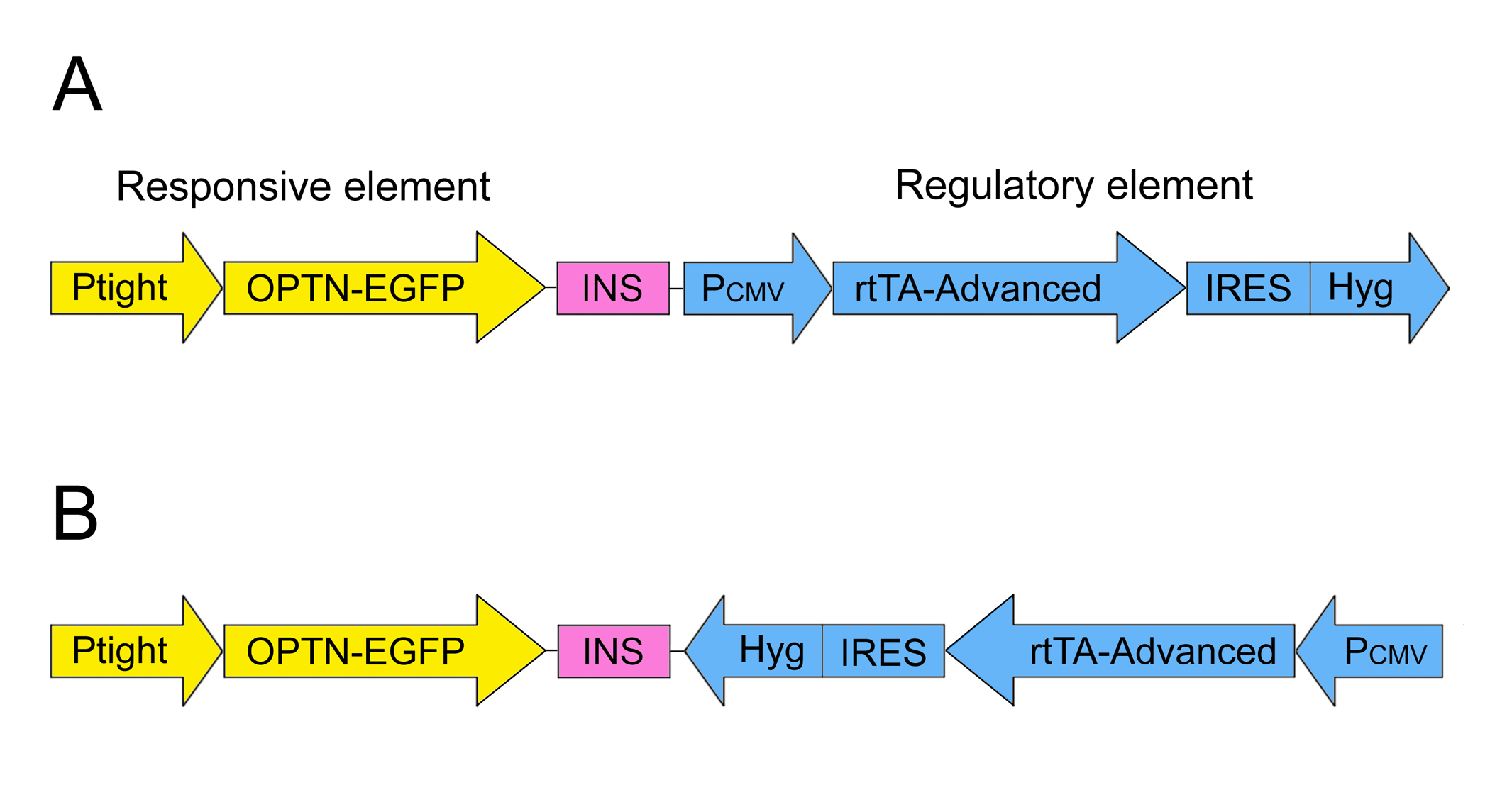

Supplement: Figure S1 — Single plasmid constructs with two expression cassettes. The two cassettes (responsive element, in yellow, and regulatory element, in blue) are separated by 5′-HS4 chicken β-globin insulator (INS, in pink). A shows the plasmid in which the regulatory element is of the same orientation as the responsive element. B shows the plasmid in which the regulatory element is of the opposite orientation as the responsive element. (7.23 MB TIF) [file pone.0009168.s001.tif]
